# Target for improvement: a cluster randomised trial of public involvement in quality-indicator prioritisation (intervention development and study protocol)

**DOI:** 10.1186/1748-5908-6-45

**Published:** 2011-05-09

**Authors:** Antoine Boivin, Pascale Lehoux, Réal Lacombe, Anaïs Lacasse, Jako Burgers, Richard Grol

**Affiliations:** 1 Scientific Institute for Quality of Healthcare, Radboud University Nijmegen Medical Centre, Nijmegen, The Netherlands; 2 Agence de la santé et des services sociaux de l'Abitibi-Témiscamingue, Rouyn-Noranda, Canada; 3 Department of Health Administration, Institute of Public Health Research of University of Montreal (IRSPUM), Montreal, Canada; 4 Département des sciences de la santé, Université du Québec en Abitibi-Témiscamingue, Rouyn-Noranda, Canada

## Abstract

**Background:**

Public priorities for improvement often differ from those of clinicians and managers. Public involvement has been proposed as a way to bridge the gap between professional and public clinical care priorities but has not been studied in the context of quality-indicator choice. Our objective is to assess the feasibility and impact of public involvement on quality-indicator choice and agreement with public priorities.

**Methods:**

We will conduct a cluster randomised controlled trial comparing quality-indicator prioritisation with and without public involvement. In preparation for the trial, we developed a 'menu' of quality indicators, based on a systematic review of existing validated indicator sets. Participants (public representatives, clinicians, and managers) will be recruited from six participating sites. In intervention sites, public representatives will be involved through direct participation (public representatives, clinicians, and managers will deliberate together to agree on quality-indicator choice and use) and consultation (individual public recommendations for improvement will be collected and presented to decision makers). In control sites, only clinicians and managers will take part in the prioritisation process. Data on quality-indicator choice and intended use will be collected. Our primary outcome will compare quality-indicator choice and agreement with public priorities between intervention and control groups. A process evaluation based on direct observation, videorecording, and participants' assessment will be conducted to help explain the study's results. The marginal cost of public involvement will also be assessed.

**Discussion:**

We identified 801 quality indicators that met our inclusion criteria. An expert panel agreed on a final set of 37 items containing validated quality indicators relevant for chronic disease prevention and management in primary care. We pilot tested our public-involvement intervention with 27 participants (11 public representatives and 16 clinicians and managers) and our study instruments with an additional 21 participants, which demonstrated the feasibility of the intervention and generated important insights and adaptations to engage public representatives more effectively. To our knowledge, this study is the first trial of public involvement in quality-indicator prioritisation, and its results could foster more effective upstream engagement of patients and the public in clinical practice improvement.

**Trial registration:**

NTR2496 (Netherlands National Trial Register, http://www.trialregister.nl).

## Background

Quality indicators can be used for setting measurable targets for improvement and ensure that quality-improvement activities tackle the most pressing areas for change [1]. Public priorities on quality improvement often markedly differ from those of clinicians and managers [2-4]. Several authors have recommended that public representatives, including patients and carers, be involved in quality-improvement activities to ensure that these efforts target their needs and expectations [5-9]. With the aging population and the growing epidemic of chronic disease, transforming the way health services are delivered for chronic disease patients is a critical focus of quality-improvement initiatives here and abroad. These changes highlight the expert and proactive role that patients, carers, and communities can play in healthcare delivery and quality improvement [10,11]. In recent years, a growing body of literature has explored the use of different methods to involve patients and the public, along with other experts, in complex healthcare policy and delivery decisions, including priority setting, health research, technology assessment, and clinical practice guideline development [12-19].

Public-involvement interventions can be classified in three broad categories: communication methods (where information is communicated *to *the public), consultation (information is collected *from *the public), and participation (information is exchanged *between *participants) [20]. To date, most of the work on patients' roles in quality improvement falls under communication and consultation methods, including public reporting of performance results [21-23]; the development of patient education material and decision aids [24]; the collection of data on patients' expectations, experience of care, and satisfaction [25-31]; or the use of open consultations in the development of quality indicators and clinical practice guidelines [4,12].

Although these involvement strategies allow patients and the public to contribute to the quality agenda, they leave several gaps unaddressed. First, the prioritisation of indicators that will be used as targets for improvement and will drive change at the clinical and management level is still largely left to panels of experts and professionals. Quality indicators can help to identify priority areas for improvement, monitor change, and report on the performance and quality of care [1]. Quality-indicator development and selection is usually based on a combination of literature review and consensus methods in which public representatives are seldom involved, despite their critical strategic importance [1]. A few examples of large-scale consensus conferences aiming at prioritising quality indicators at the national or international level have included patient and public representatives, but these initiatives have never been formally evaluated [32-34].

A second gap in current involvement strategies is that consultations on patients' experience of care and satisfaction often focus on those dimensions of care that are easier to be appraised by patients, such as interpersonal communication and access, as opposed to other clinical and organisational aspects of care [4]. Also, patients involved through communication and consultation methods tend to appraise and judge quality in relation to their own individual care, without consideration of existing research evidence, the competing needs of different users in the community, and the constraints of available resources and services. As a result, health professionals, policy makers, and the public often operate in different and separate worlds in relation to quality improvement [35,36].

In response to those limitations, there is a growing call for public-involvement methods that allow for active participation and deliberation between stakeholders with different expertises and knowledge [37]. Public deliberation is a 'means by which the public can influence the generation of data and the derivation of the policy options as well as discussing acceptable decisions, thus, taking account of public as well as expert knowledge [38]'. Deliberation is expected to result in (a) mutual learning between participants; (b) the generation of options that are formed on the basis of broader perspectives, interests, and information; and (c) the formation of solutions that most people involved in the deliberative process can find acceptable [17,39].

Consultation, participation, and communication methods rest on different theoretical assumptions and methods. In the academic literature, a methodological and paradigmatic divide tends to separate proponents of consultation strategies (based on the collection of data from population surveys and other epidemiological methods) and proponents of participation methods that rest on deliberative theory and political sciences [39,40]. Similarly, communication experts tend to focus their work on methods to present information and evidence to individual patients and public members in order to support healthcare choices, behaviour change, and public accountability [24,41]. As a result, mixed public-involvement strategies have rarely been tested, although a number of quality-improvement organisations do combine these different strategies in practice [12].

Many doubts remain regarding the feasibility and impact of public involvement in quality improvement [14,42-44]. To date, most empirical research on public involvement in healthcare has studied the process of involvement and its perception by participants (*e.g.*, whether public representatives are satisfied with the experience and feel that deliberations were fair); no study has assessed the impact of public involvement in quality-indicator prioritisation [14]. A recent knowledge synthesis identified many barriers to the development of effective involvement programs, including the following: the lack of evidence on public-involvement effectiveness, concerns that public involvement may often be tokenistic and is unlikely to influence group decision making, the technical complexity of the task, the difficulty in identifying and recruiting public members who are competent and representative, the gap between professional and public perspectives, and the feasibility of public-involvement interventions in terms of time constraints and cost [45].

Our goal is to assess the feasibility and impact of public involvement on quality-indicator prioritisation. Our specific aims are the following:

1. Evaluate the impact of public involvement on:

a. quality-indicator choices and agreement with public priorities (primary outcome);

b. decision makers' intention to use the indicators for quality improvement.

2. Identify factors that explain the effectiveness of the public-involvement program.

3. Estimate the costs of involving the public in quality-indicator prioritisation.

Our main hypothesis is that public involvement will result in quality-indicator choices that better agree with public priorities.

## Methodology

### Project overview and design

**Figure 1:**
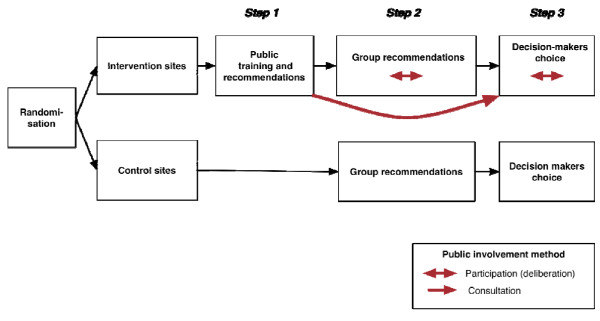
**Project overview**. In intervention sites, public representatives are involved in quality-indicator prioritisation through consultation and participation methods, while prioritisation in control sites does not involve public representatives.

We will conduct a cluster randomised controlled trial that will assess the impact of public involvement on quality-indicator choice and intended use (Figure 1). A cluster design is warranted because of our interest in group decision making. In preparation for the trial, we have developed a 'menu' of validated quality indicators based on a systematic review of the literature and expert consultation. We also pilot tested our intervention and instruments. Participants (public representatives, clinicians, and managers) will be recruited from six participating sites, which will be randomised in intervention (quality-indicator prioritisation with public involvement) and control sites (without public involvement).

Quality-indicator prioritisation will be conducted in three steps. In step 1, public representatives will have a one-day training session to familiarize themselves with the proposed indicators and will be asked to make individual recommendations on indicator choice. In step 2, public representatives will participate in a one-day deliberative meeting with clinicians and managers to agree on five group recommendations. In step 3, individual and group recommendations will be fed back to decision makers, who will choose the indicators to be selected as local targets for improvement and discuss actions to support their use in clinical and management practices.

Public-involvement methods in intervention sites will combine participation (deliberation between public representatives, clinicians, and management) and consultation methods (public priorities collected at the training meeting will be fed back to decision makers). Quality-indicator prioritisation in control sites will only involve clinicians and managers.

Data on quality-indicator priorities will be collected from participants at each meeting. Decision makers' intentions to use the selected indicators for quality-improvement purposes will also be collected at the end of the step 3 meeting. This study was approved by the Université du Québec en Abitibi-Témiscamingue ethics committee. The following section will describe in detail the process of intervention development and pilot testing, as well as the protocol of the trial that will be used to assess the intervention's impact.

### Study setting

Abitibi-Témiscamingue is one of the largest administrative regions of Québec, Canada, with a population of 145,886 people, including 6,500 people (4.5%) from First Nations communities. The economy of the region is centered around the mining and wood industry. Most of the population is francophone and 4% have English as their first language [46]. The Regional Health Authority of Abitibi-Témiscamingue (Agence de la santé et des services sociaux de l'Abitibi-Témiscaminge [ASSSAT]) is responsible for coordinating the services in the region. The region is divided into six local service networks, each one under the responsibility of a local health authority (Centre de santé et de services sociaux [CSSS]). The six local health authorities cover rural territories of a few thousand people with basic community care and medium-size towns of approximately 40,000 people with specialised hospital care. Local Health Authorities are responsible for ensuring access to health and social services for the population in its territory through direct service delivery and agreements with partner organisations in its local services network (medical clinics, community organisations, specialist services and hospitals, etc.) [47]. Most family physicians providing primary care services in the region are organised in family medicine groups (Groupes de Médecine Familiale [GMFs]), a group of family physicians working in close collaboration with nurses in an environment that fosters providing family medicine to registered individuals. Family physicians in the region cover many secondary care services (*e.g.*, emergency room, hospital care, obstetrical care, intensive care unit). Each local health authority is more than 100 km from another local health authority and serves a rather captive population that receives most of its care within its own community.

Since 2005, the ASSSAT has been implementing a regional chronic disease prevention and management program based on the integration of public health approaches and clinical services for chronic disease prevention and management, the promotion of interdisciplinary work, collaboration with community organisations, self-care support, and case management [48]. Modelled on the Expanded Chronic Care Model [49], this regional program targets the prevention and management of four chronic conditions (diabetes, chronic obstructive lung disease, ischemic heart disease, and heart failure) but also supports broader structural changes and integration within local health authorities and their local services network partners. Adaptation of the regional program to local priorities and context has been encouraged since the beginning of the program. An implementation evaluation of the program conducted in 2008-2009 concluded that the development and use of quality indicators could help support change and quality improvement at the local level [50]. The target for improvement trial was developed and integrated within the overall implementation strategy of the ASSSAT regional chronic disease prevention and management program. The study will be conducted among the six local health authorities of the region.

### Identification of quality indicators

We used a systematic process to develop a menu of quality indicators on chronic disease prevention and management that would be valid, relevant within the context of primary care in Canada, and measurable using existing information systems. To be included, the identified indicator had to:

1. relate to the prevention or management of chronic diseases, defined as health conditions requiring ongoing management over a period of years or decades [51]. We included generic indicators applicable to any chronic disease and disease-specific indicators related to the prevention and management of type 2 diabetes, chronic obstructive pulmonary disease, coronary heart disease, or heart failure;

2. measure an element of practice structure, process, or outcome for which there is evidence or consensus that it can be used to assess the quality, and hence effect change, in the quality of care provided [1];

3. have been cited in a peer-review publication that either described its development process, assessed its psychometric properties, or used it for research and evaluation.

We grouped our indicators into five quality domains: access, integration, technical quality of prevention and clinical management, interpersonal care, and outcomes. Our classification was developed from a concept analysis of existing quality-domain frameworks [2,3,33,34,52-59] and rested on operational definitions of primary care attributes developed by Canadian experts [59].

**Figure 2:**
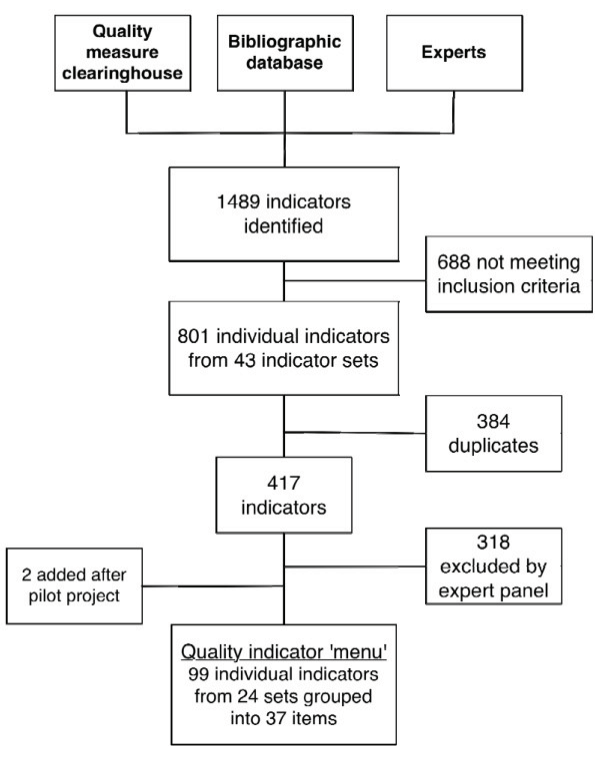
**Systematic review of quality indicators flowchart**. Systematic review and selection of existing validated quality indicators for chronic disease prevention and management in primary care.

Figure 2 summarises the indicator identification and selection process. We first conducted a systematic search for quality indicators from the National Quality Measure Clearinghouse [57]^1^and bibliographic databases (MEDLINE, PsycINFO, HTA Database, NHS Economic Evaluation Database, EconLit, Business Source Premier, Health and Psychosocial Instruments)^2^, as well as through contact with experts and key informants and hand-searching of reference from relevant papers.

We identified a total of 1489 individual indicators. 801 indicators met our inclusion criteria. We extracted each included individual indicator and built a quality-indicator database. Two independent researchers, including the principal investigator, identified and removed duplicates. When multiple related clinical care indicators were present, we chose indicators that were developed in Canada or that were most closely aligned with current Canadian clinical practice guidelines [60-65]. We presented the remaining list of individual indicators to a panel of five experts (two physicians, two health managers, and an information specialist) who shared collective expertise in the clinical and organisational aspects of chronic disease management and knowledge of the clinical context and the available information systems. Expert panel member independently rated each indicator based on relevance and measurability. Expert panel members met twice to agree on the final list of indicators.

Primary care delivery in Canada is largely provided by family physicians, but allied health professionals, such as primary care nurses and nurse practitioners, are playing an increasing role in this area. To reflect these system characteristics, we adapted the wording of some indicators by changing 'regular doctor' to 'family doctor' or 'regular primary healthcare provider', in accordance with current Canadian indicators [66]. We translated the selected indicators in French and wrote a plain language description of each. Our expert panel validated the indicator translation and description. Subscales of individual questionnaires (*e.g.*, the Primary Care Assessment Survey continuity domain [67]) and disease-specific clinical indicators (e.g. clinical management of type 2 diabetes) were grouped together as individual menu items.

The proposed indicator menu was tested for comprehensiveness and relevance with a group of public representatives and professionals in our pilot project (described below). The final menu of indicators is composed of 37 menu items (Table 1). The complete description of each indicator and a reference to the original indicator set is included in Additional file 1.

**Table 1:** Menu of quality indicators

Access	
1. Perceived difficulty to obtain an appointment	2. Primary healthcare organisation's opening hours

3. Access for disabled people	4. Family physicians accepting new patients

5. Medication and treatment cost	6. Language barriers

7. Phone access to a primary care provider	

**Integration**	

8. Coordination among healthcare organisations	9. Electronic communications

10. Primary care registries for chronic conditions	11. Perceived continuity of care

12. Team work and interdisciplinary care	13. Links with community organisations

**Technical quality of prevention and clinical management**	

14. Physical activity counselling	15. Healthy eating counselling

16. Tobacco counselling	17. Influenza vaccination

18. Hypertension screening	19. Perceived technical quality of care

20. Clinical management of type 2 diabetes	21. Clinical management of coronary heart disease

22. Clinical management of chronic obstructive pulmonary disease (COPD)	23. Clinical management of heart failure

**Interpersonal care**	

24. Self-care support	25. Patient participation in clinical decision making

26. Respect and empathy	27. Time available during the consultation

28. Trust toward primary care provider	29. Stress and responsibilities at work and at home

**Outcomes**	

30. Fruit and vegetable consumption rate	31. Smoking rate

32. Physical activity rate	33. Blood pressure control

34. Perceived self-efficacy	35. Hospitalisation for ambulatory-care-sensitive conditions

36. Emergency room visit for ambulatory-care-sensitive conditions	37. Quality of life

### Development of the intervention and pilot testing

The development, pilot testing, and refinement of the intervention followed a structured framework for the design and evaluation of complex interventions in health [68]. Our public-involvement intervention development is based on best-practice recommendations for public involvement in healthcare [5,12,13,69-72] and quality-indicator development [33,73-75]. We sought to use a public-involvement strategy that combined consultation and participation methods. The consultative component aims at collecting public recommendations from a broad and diverse group of public representatives. The participation component aims at supporting deliberation among clinicians, managers, and public representatives to foster mutual learning, respectful disagreement, consensus building, and the emergence of a collective perspective on quality improvement [20,39]. Our quality-indicator prioritisation process is based on the RAND appropriateness method, which combines a systematic review of existing indicators, an individual rating of indicators by a Delphi procedure, and a face-to-face deliberation and rerating of indicators using nominal group technique [76].

Research questionnaires were pretested with 21 people before being used in our three pilot meetings. We pilot tested the format of step 1 and step 2 meetings in the region of Lanaudière (Québec), 500 km away from the participating sites. The northern part of this region has sociodemographic and health system characteristics that are similar to those of the region of Abitibi-Témiscamingue, thus allowing us to test the feasibility of the intervention without contaminating our study sites. Nineteen participants (nine public representatives, eight clinicians and managers) participated in the step 1 and step 2 pilot meetings in January and February 2010. We pilot tested our decision makers' meeting (step 3) with 10 participants (two public representatives, eight managers and clinicians) from the Regional Health Authority of Abitibi-Témiscamingue at the end of September 2010. Two researchers were present during each pilot meeting and took observation notes. A structured debriefing session was held with participants at the end of each pilot meeting to identify what worked and what did not and to collect suggestions for improvement. We held debriefing meetings with our team to adjust the intervention format and data collection instruments based on participants' comments and observations.

As a result of our pilot testing, we adapted our intervention and instruments and decided to:

1. clarify participants' responsibilities, by developing a detailed written task description;

2. introduce the menu of indicators to public representatives during the training session;

3. develop structured recruitment documents with explicit representation criteria to facilitate the identification of public representatives from different socioeconomic backgrounds;

4. invite more public representatives and physicians in step 2 meetings to deal with potential attrition;

5. prepare a seating plan to facilitate interactions between public representatives, clinicians, and managers;

6. develop structured prompts and suggestions to support the group deliberation process and enable participants to complete the task more effectively;

7. add two new items to the indicator menu on stress and collaboration with community organisations, in response to public representatives and professionals' suggestions;

8. use videorecording rather than audiorecording to better capture social interactions among participants;

9. use color coding and ranking of step 1 and step 2 reported recommendations, to facilitate their communication to decision makers in step 3 meetings;

10. clarify the regional health authority's expectations toward indicator use.

### Recruitment and randomisation of the participating sites

The local health authorities' Chief Executive Officers (CEOs) and GMF medical directors from all six territories of Abitibi-Témiscamingue agreed to participate in the study (response rate = 100%). Site randomisation will be done after the recruitment process of individual participants is completed, using a random allocation software [77]. Randomisation will be carried out by one of the researchers, with two independent observers present, and will be concealed to the professionals in charge of recruitment, the group facilitator, and participants until the end of the step 1 meeting (see Control section below).

### Individual participants' recruitment

Within each local health authority participating in the study, we created recruitment teams who are responsible for identifying public representatives, clinicians, and managers interested participating in the study. Each local recruitment team includes a member of the CSSS user committee, the manager in charge of the chronic disease program, and the medical director of the family medicine group. Local health authorities' CEOs will also be solicited to identify the managers and clinicians who will act as decision makers. Local recruitment teams will identify potential participants by purposive sampling and the snowballing technique, using our inclusion and representation criteria described in Table 2[78]. We seek to recruit clinicians and managers who are closer to healthcare delivery to participate in the step 2 meeting (group recommendations) and senior-level managers and professional council representatives for step 3 (decision makers' meeting), allowing for overlap between both meetings.

**Table 2:** Inclusion and representation criteria

Category of participant	Inclusion/exclusion criteria	Representation criteria
**Public representatives** Steps 1, 2, 3 meetings (Target: 90 participants)	1) Adult with or without a chronic condition 2) Be competent to share opinions with others 3) Not be currently or previously working as a clinician or healthcare manager	Age, gender, employment, and health status (healthy adults without chronic disease, patients with uncomplicated chronic disease, patients with complex chronic conditions)

**Clinicians and managers** Step 2 meeting (Target: 72 participants)	1) Work as a clinician or manager in relation with the prevention or management of chronic diseases 2) Work within the catchment area of a participating health authority 3) Be competent to share opinions with others	Include a minimum of two primary care physicians, one manager familiar with the chronic disease program and existing information systems, and a balanced mix of clinicians and managers involved in chronic disease prevention and management

**Clinicians and managers** Step 3 decision makers' meeting (Target: 60 participants)	1) Be identified by the local health authority's CEO to advise him/her on the choice of quality indicator 2) Be a member of the board or professional council of the local health authority or family medicine group	Include the CEO or his/her representative, as well as one physician; the identification of other key decision makers is left to the CEO's discretion

CEO = chief executive officer.

For the purpose of our study, a public representative can include any adult targeted by the regional chronic disease prevention and management program who is not a healthcare professional or employee. This includes healthy adults, carers, and patients with chronic conditions. Interested individuals will be given a written description of the project and a 'job profile', explicitly stating that we are looking for people who represent a broad range of backgrounds and personal experiences and who are willing to work collaboratively with other public representatives, clinicians, and managers (Table 2). Identification of public representatives through local recruitment teams allows us to reach public members who have perceived legitimacy within their own community and who are interested in the issues discussed [79]. A research assistant will contact potential participants, confirm their eligibility criteria and interest/availability for participating in the study, and collect basic sociodemographic characteristics. The research team will select participants based on the representation criteria described in Table 2.

### Description of the intervention

The intervention is composed of three one-day meetings (step 1, step 2, and step 3) that aim at prioritizing local quality indicators. The Regional Health Authority expects that the selected indicators will be used to support continuous quality improvement of chronic disease prevention and management (rather than for external control or benchmarking), and each local health authority will be allowed to select its own indicators. The selected indicators will be integrated in the regional accountability contracts signed with each local health authority. Table 3 summarises the topics addressed in each intervention meeting, and their content is described in detail below.

**Table 3:** Intervention meetings' content

Meetings	Participants	Content
**Step 1: Public representatives' training and recommendations**	Public representatives (Target: 15/site)	• Participants' discussion on positive and negative experience in relation to quality of care • Information on chronic disease and local prevention and management services • Explanation of the indicator menu and data collection on baseline public recommendations

**Step 2: Group recommendation**	Clinicians and managers (Target: 9/site) and public representatives (Target: 6/site)	• Individual baseline prioritisation • Deliberation on indicator choice ○ Block 1 (Structure: access and integration) ○ Block 2 (Process: technical quality and interpersonal care) ○ Block 3 (Outcome indicators) • Final group recommendation and individual recommendations

**Step 3: Decision makers' meeting**	Clinicians and managers (Target: 10/site) and public representatives (Target: 2/site)	• Expectations from the Regional Health Authority on quality-indicator choice and use • Presentation of recommendations issued in step 1 and step 2 meetings • Deliberation on indicator choice and implementation • CEOs summarise decisions and foresee actions for each local health authority

CEO = chief executive officer.

### Step 1: public representatives' training and recommendations

The step 1 meeting aims to train public representatives and to collect their individual recommendations for local quality improvement. Public representatives (target: 15 per site) will meet with the moderator for a one-day meeting. Participants will be asked in turn to reflect and share their experiences with and expectations toward quality of care, will receive background information on chronic disease and on existing prevention and management services in their community, and will receive explanations on the proposed quality indicators. At the end of the meeting, public representatives will individually prioritise the quality indicators and identify five indicators that they recommend as local targets for improvement (public baseline recommendations).

### Step 2: group recommendations

In the step 2 meeting, public representatives, clinicians, and managers will deliberate together to agree on five local group recommendations. We will aim to recruit a total of 15 participants in each group (nine clinicians and managers and six public representatives). We will recruit public representatives from step 1 participants, based on their availability, interest, and natural attrition. If more people volunteer, the research team will select candidates based on our representation criteria to ensure a balanced representation of age, gender, employment, and health status (Table 2).

Group rating and deliberation on quality-indicator prioritisation will be done in four steps: (1) participants prioritise indicators individually at the beginning of the day; (2) feedback on individual responses is given to the whole group; (3) participants deliberate as a group on the indicators' pros and cons; (4) if consensus on group recommendations cannot be reached, the moderator asks participants to vote. At the end of the day, participants will be asked to agree on five indicators that they recommend using as targets for improvement in their territory (group recommendation). They will also be asked to record five indicators that they recommend individually. We will explain to the participants that it is not necessary for everybody to agree with the final group recommendations, as long as everyone can 'live with' the compromise or consensus reached by the group.

### Step 3: decision makers' meeting

In the step 3 meeting, decision makers identified by the local health authority's CEO will choose which indicators to use as local targets for improvement and identify actions to implement these indicators in clinical practice and management. While step 1 and step 2 meetings will be held locally within each participating site, we will hold one semiregional step 3 meeting that will bring together decision makers from all intervention sites, and another semiregional meeting with all control sites. A semiregional format will allow us to involve senior directors from the Regional Health Authority and send consistent messages across all sites regarding the Regional Health Authority's expectations.

Local and regional recommendations developed in steps 1 and 2 meetings will be presented to decision makers. Individual recommendations will be communicated to decision makers by reporting the rank of each indicator, calculated from the proportion of participants who recommended each indicator. Group recommendations and individual recommendations will be color-coded to facilitate their identification by decision makers. Recommendations will be discussed in small-group deliberation sessions within each site. At the end of the meeting, each local health authority's CEO will summarise the decisions and actions proposed within his/her own territory. A Regional Health Authority representative (RL) will be present to explain the quality indicator expected use, describe the professional and technical resources that will be available to support quality-indicator implementation, and answer questions.

Public involvement in the step 3 meeting will combine consultation and deliberation methods. Decision makers will receive written feedback about individual recommendations made by public representatives in step 1 meetings (consultative component). Public representatives who participated in step 1 and step 2 meetings will also be invited to attend the meeting (target: two participants/site) to answer decision makers' questions and assist them in their choice (participation component).

### Moderator

A professional moderator (JL) with previous experience in communication and group facilitation will moderate all step 1 and step 2 meetings and will also facilitate the step 3 plenary sessions. In the step 3 meeting, two additional moderators will facilitate small-group deliberation among decision makers from each site. All moderators attended our pilot meetings and participated in a preparation session to develop an animation grid, agree on solutions to potential pitfalls, and develop prompts to guide discussions. The moderators will be responsible for welcoming participants, establishing ground rules with them, ensure fair participation, and facilitate deliberation and agreement on the proposed indicators and actions. A member of the research team (AB) will attend all meetings, present the project and the proposed indicators, and answer technical questions.

### Control

In control sites, quality-indicators prioritisation will be done by clinicians and managers only, following the format described for the above step 2 and step 3 meetings. Public representatives will not be involved in quality-indicator prioritisation.

For research purposes, we will also conduct step 1 meetings in all control sites to collect data on local public recommendations (see the Data Collection and Analysis sections below). The format and content of the step 1 meeting will be identical in control and intervention sites. The moderator and participants will be blinded to their allocation until the end of the meeting. We will present the results of this public consultation to control sites' decision makers at the very end of the step 3 meeting, after we collect all trial outcome data on quality-indicator choice and intended use.

The six participating sites are more than 100 km apart from one another, clinicians and managers have rare contact among themselves, and they serve rather captive populations who receive most of their care within their community, thus minimising the potential for contamination across intervention and control groups. We will ask all participants to respect the confidentiality of discussions and not to share any information in between meetings. We will assess for potential contamination among participants in all meetings.

### Data collection

Table 4 describes the questionnaires that will be used for data collection. Specific data collection instruments are described in detail below. Research questionnaires were pretested with 21 persons, before being used in our three pilot meetings (described above).

**Table 4:** List of questionnaires

#	Timing	Respondents	Data collected
Q1	Beginning of step 1	Public	Public representatives' sociodemographic data (age, gender, ethnic group, language, education, socioeconomic status, health status, health services use, prior attitude toward public involvement)

Q2	End of step 1	Public	Quality-indicators prioritisation (public baseline priorities)

Q3	End of step 1	Public	Participants' evaluation of the step 1 meeting

Q4	Step 2 and step 3 meetings	Clinicians and managers	Clinicians and managers' sociodemographic data (age, gender, ethnic group, language, education, socioeconomic status, professional role, prior attitude toward public involvement)

Q5	Beginning of step 2	Clinicians and managers	Quality-indicator prioritisation (clinicians and managers' baseline priorities)

Q6	End of step 2	Clinicians, managers, and public representatives	Quality-indicator prioritisation (postdeliberation priorities)

Q7	End of step 2	Clinicians, managers, and public representatives	Participants' evaluation of the step 2 meeting

Q8	End of step 3	Clinicians, managers, and public representatives	Quality-indicator prioritisation, attitude and intention to use the selected indicators for quality improvement (decision makers' choice and intention to use)

Q9	End of step 3	Clinicians and managers (control sites only)	Quality-indicator prioritisation (postconsultation priorities); this questionnaire is completed after we collect data on decision makers' choice and intention to use, and after we present results of public consultation to control sites

Q10	End of step 3	Clinicians, managers, and public representatives	Participants' evaluation of the step 3 meeting

### Quality-indicator prioritisation

**Figure 3:**
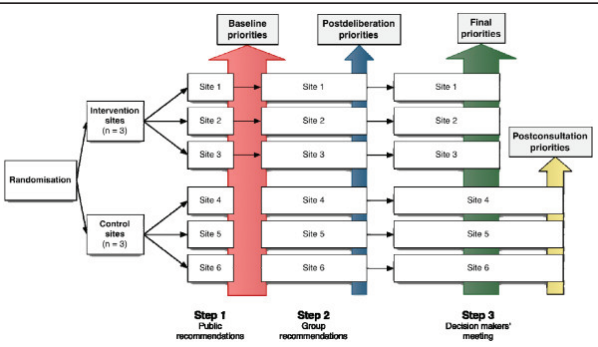
**Data collection on quality-indicator prioritisation**. Participants' priorities will be collected from each site at baseline and after each meeting.

Our primary outcome is the comparison of indicator choice and agreement with public priorities between intervention and control groups. Data on quality-indicator prioritisation will be collected at baseline and at the end of each meeting (Figure 3). In order to collect public baseline priorities from all participating sites, we will hold step 1 meetings with public representatives from the six participating sites. Clinicians and managers' baseline priorities will be collected at the beginning of the step 2 meeting. Postdeliberation priorities will be collected at the end of the step 2 meeting. Decision makers' choice and final priorities will be collected at the end of the step 3 meeting. We will also collect postconsultation priorities from control site participants at the end of the step 3 meeting, after we collect data on decision makers' choice and intention to use and present results of public consultation. Postdeliberation and postconsultation priorities will be used for process evaluation purposes to assess the contribution of each component of the intervention.

The questionnaire on quality-indicator prioritisation includes the menu item title, a description of the indicator under each item (*e.g.*, 'percent of family physicians who accept new patients'), as well as the source of information (patients' charts, administrative data, or survey) and original reference (Additional File 1). At the end of each questionnaire, participants are asked to prioritise five quality indicators ('indicate the five indicators that you believe are the most important to improve chronic disease prevention and management in your territory') and to rank these five indicators in order of importance [80].

In step 1 and step 2 meetings, a research team member (AB) will read each item individually and answer questions. Participants in these two meetings will be asked to rate each indicator according to its perceived importance and feasibility, using a Likert scale from 1 to 9 [2]. In step 3, participants will be sent the indicator by mail before the meeting. Decision makers will be asked to prioritise their five most important indicators after they receive feedback on individual and group recommendations.

### Decision makers' intention to use the selected indicators

The questionnaire on decision makers' attitude and intention toward indicator use will be completed by all participants in the step 3 decision makers' meeting, after decision makers agree on which indicators they will select as targets for improvement for their territory. We have developed this questionnaire from known predictors and instruments used to measure the likely adoption of quality indicators and health innovations [75,81-85]. The questionnaire consists of 11 items covering decision makers' attitude toward selected quality indicators (importance, feasibility, credibility, group consensus) and their intention to use and report on the selected indicators for quality-improvement purposes. Each item is scored on a 7-point Likert scale.

### Cost analysis

In order to estimate the financial cost of public involvement in quality-indicator prioritisation, a cost analysis will be conducted. In this type of analysis, the costs of an intervention are presented in a disaggregated form [86]. We will adopt the perspective of the intervention sponsor and report on the marginal financial costs of public involvement, including the costs of public representatives' recruitment, training, financial compensation, group facilitation, administrative support, meals, and didactic material. The average costs per site will be estimated based on actual project expenses.

### Process evaluation

In the context of trials, process evaluation can be used to explain the study's results [87,88]. Our process evaluation will focus on understanding the effects of the intervention and the mechanisms that underlie change. A multiple case study will be used for this analysis, capitalising on natural intersite variations. Our analysis will be guided by group process and deliberative theory to explore how public involvement influences the content of deliberation and the social interactions among participants [39,89-91]. Data collection will be carried out using standardised questionnaires, direct observation of all meetings by two independent nonparticipant observers, and video recording of all meetings. A group debriefing session will be held with participants at the end of each meeting. A standardised self-administered evaluation questionnaire will also be distributed at the end of each meeting, based on an existing deliberation assessment questionnaire [92]. The evaluation questionnaire is composed of 22 items divided into five domains covering (1) roles, procedures, and objectives; (2) meeting facilitation and support; (3) information received; (4) participants' interaction; and (5) overall satisfaction. Each item is scored on a 7-point Likert scale. The observers and moderators will hold a debriefing session among themselves immediately after each meeting to share observations.

### Statistical analysis

Descriptive statistics will be used to summarise the characteristics of the study population and assess the comparability of intervention and control groups, as well as to summarise data on quality-indicator choice, intended use, and on the marginal costs of the intervention.

We will descriptively report which quality indicators are selected as targets for improvement within each site at the end of the trial and calculate the proportion that are in agreement with local public baseline priorities (ranks 1 to 5). Individual quality-indicator priorities will be analysed as a dichotomous measure by reporting the proportion of participants who selected each indicator as part of their five priorities and by calculating its rank (rank 1 = indicator selected by the greatest proportion of participants). Agreement with public priorities will be analysed by calculating the correlation between professionals (clinicians and managers) and public priorities at baseline and at the end of the trial (primary outcome). Cluster randomisation leads to a reduction of effective sample size and can give spurious statistical results if it is not accounted for properly [93,94]. We will check the data to assess the level of clusterisation within study sites and use appropriate cluster-level analysis (*e.g.*, multilevel modelling) if necessary. We will also compare decision makers' intention to use the selected indicators and participants' satisfaction between intervention and control sites. Statistical significance will be assumed at *p *< 0.05 (two-tailed test) for all tests.

### Sample size

Our sample size calculation is based on pragmatic considerations and takes into consideration the maximum number of available sites/clusters in the region (n = 6 sites) and the maximum number of recommended participants in small-group deliberation meetings (n = 15 participants per meeting). We aim to recruit a total of n = 90 public representatives, n = 72 clinicians and managers for the step 2 meeting, and n = 60 senior managers and professional council representatives for the step 3 meeting. We will allow for overlap between clinicians and managers participating in step 2 and step 3 meetings.

Abelson and colleagues note that small sample sizes are hard to overcome in studies of public participation in healthcare as 'deliberation decision-making dictates small groups' [13]. We expect that the power of our study will be further decreased by the cluster nature of the trial, although we are currently unable to estimate the magnitude of this effect due to the absence of prior trials of public involvement in quality-indicator prioritisation and unknown intra-cluster coefficients for our outcome of interest [93].

### Integrated knowledge translation and postrandomisation follow-up

We are following an integrated knowledge-translation plan throughout the trial preparation and implementation, where knowledge users are directly involved in strategic aspects of research and knowledge production [95]. This study is embedded in a larger implementation strategy of the regional integrated chronic disease prevention and management program [50]. Our team is pursuing two core objectives in this project: (1) to support chronic disease prevention and management through the selection and use of quality indicators that will be used as local targets for improvement (practice component) and (2) to assess the impact of public involvement on quality-indicator prioritisation and intended use (research component). Within this project, partnership between decision makers and researchers will be an ongoing process throughout the cycle of knowledge production and use. At each stage of the intervention, we will collaboratively (a) plan the initial 'blueprint' of the intervention; (b) pilot test it; (c) 'lock in' the final format of the intervention for its implementation in the trial; and (d) collect, analyse, and communicate knowledge to researchers and decision makers.

Our target knowledge users include clinicians, managers, and public representatives from local health authorities and the Regional Health Authority, as well as provincial and national organisations involved in indicator use and quality improvement. The principal investigator (AB) will act both as a researcher (IQ Healthcare) and as a medical advisor for the Regional Health Authority (ASSSAT) and will be responsible for facilitating the interaction between decision makers and researchers on the project. A member of the Regional Health Authority's board of directors (RL) has been included in all aspects of the study design and research. Key aspects of the study protocol were presented and discussed with the CEOs of all participating local health authorities, medical directors of family medicine groups, the Regional Health Authority's board of directors, as well as with local and regional users' committees and population forums. The project was also presented to the Québec provincial government in February 2010 [96]. Representatives from AETMIS, a provincial organisation that has received the mandate from the provincial Ministry of Health to develop quality indicators for primary care improvement, have also partnered with us on the project.

Gibbens argues that research conducted in the context of application has the potential to increase the relevance and impact of the knowledge produced and to foster its use and implementation in practice [97]. The Regional Health Authority of Abitibi-Témiscamingue is committed to supporting indicator implementation and use after the completion of the study to support the improvement of chronic disease prevention and management. Professional and technical resources will be made available regionally to support indicator use. Follow-up on quality-indicator use will be integrated in the regional director-generals meeting, as part of a statutory point on chronic disease prevention and management.

## Discussion

To the best of our knowledge, this study is the first trial of public involvement in quality-indicator prioritisation [14]. It tackles important knowledge gaps on how members of the public, including patients and carers, can be effectively involved in strategic aspects of quality improvement. A strength of the study is the systematic approach that was used to develop and refine the public-involvement intervention, based on existing frameworks for the development and testing of complex health interventions. Our pilot project provided important insights on how to engage public representatives more effectively. The testing of this intervention in a real-world prioritisation context has the potential to increase the external validity of findings and test the feasibility of the intervention in practice. A limitation of the study is our small effective sample size, given the cluster nature of the trial and restrictions regarding the maximum number of sites and individual participants that can be recruited for a deliberative intervention. We nonetheless expect that this trial will provide important knowledge into the feasibility, process, and effectiveness of public involvement in quality-indicator prioritisation, thus fostering upstream engagement of patients and the public in clinical practice improvement.

## Declaration of competing interests

The authors declare that they have no competing interests.

## Authors' contributions

AB, PL, RL, JB, and RG designed the study. AL contributed to the development of the research questionnaires and the statistical analysis plan. All authors revised the protocol critically for important intellectual content and approved its final version for publication.

## Supplementary Material

Additional file 1 **Description of the quality indicators used in the Target for Improvement Trial**. Additional file 1 includes the detailed description of the final 37-item 'menu' of quality indicators used in the trial, with references to the original indicator sets. Click here for file
