# Supplementary material for: Target for improvement: a cluster randomised trial of public involvement in quality-indicator prioritisation (intervention development and study protocol)
Source: Implement Sci. 2011 May 9;6:45. doi: 10.1186/1748-5908-6-45 (PMC3118228; doi:10.1186/1748-5908-6-45)
Supplement: Additional file 1 — Description of the quality indicators used in the Target for Improvement Trial. Additional file 1 includes the detailed description of the final 37-item 'menu' of quality indicators used in the trial, with references to the original indicator sets. [file 1748-5908-6-45-S1.DOC]

# Additional file 1: Description of the quality indicators used in the Target for Improvement Trial

The following quality indicators on chronic disease prevention and management will be presented to all study participants in the Target for Involvement Trial. The indicator “menu” contains 37 items including structure, process, and outcome indicators. The indicators are divided in five quality domains (access, integration, technical quality of preventive and clinical care, interpersonal relationship, and health outcomes). Sub-scales of individual questionnaires (e.g. the Primary Care Assessment Survey continuity domain) and disease-specific clinical indicators (e.g. clinical management of type 2 diabetes) were grouped together as individual menu items.

In Step #1 and #2, each indicator will be read aloud by a member of the team at the beginning of the meeting. In Step #3, the menu will be mailed to Decision-Makers prior to the meeting. The group moderators received training to help them understand the indicator menu, and a member of the research team will be available in all meetings to answer technical questions.

This “indicator menu” forms the basis for questionnaire #2-5-6-8-9 on quality indicator prioritization, as well as for written documents on individual and group recommendations distributed to decision-makers in Step #3 meeting.

**ACCESS**

| **N^o^** | **Quality indicator** | **Source** | **Reference** |
| --- | --- | --- | --- |
| **1** | **Perceived difficulty to obtain an appointment:**   - Perceived delay to obtain an appointment with a primary care provider for minor problems and routine care - Perceived difficulty to obtain an appointment with a primary care provider[[1]](#footnote-2) | Patient reported questionnaire | Canadian Institute for Health Information [1]  Primary Care Assessment Survey [2] |
| **2** | **Primary health care organization’s opening hours :**  Average primary care organizations’ opening hours in the evening (after 17h) and weekends | Administrative data | Canadian Institute for Health Information [1] |
| **3** | **Access for disabled people**  Primary care services are physically accessible for disasbled people | Administrative data | Quality indicators for general practice [3] |
| **4** | **Family physicians accepting new patients :**  % of family physicians accepting new patients | Administrative data | Canadian Institute for Health Information [1] |
| **5** | **Medication and treatment cost :**   - Do you ever skip medication or treatments because they are too expensive? - How would you rate the amount of money you pay for medication & other prescribed treatments? | Patient reported questionnaire | Primary Care Assessment Survey [2] |
| **6** | **Language barriers :**  % of patients/clients who experienced language barriers when communicating with their regular primary care provider, over the past 12 months | Patient reported questionnaire | Canadian Institute for Health Information [1] |
| **7** | **Phone access to a primary care provider :**  Possibility to obtain information and advices over the phone with a primary care professional when a visit is not necessary or difficult[[2]](#footnote-3) | Administrative data | Quality indicators for general practice [3] |

| **INTEGRATION** |
| --- |

| **N^o^** | **Quality indicator** | **Source** | **Reference** |
| --- | --- | --- | --- |
| **8** | **Coordination among health care organizations:**  % of primary health care organizations who currently coordinate client/patient care with other health care organizations (eg. hospital, specialized clinics) using standardized clinical protocols or assessment tools | Administrative data | Canadian Institute for Health Information [1] |
| **9** | **Electronic communications:**  % of primary care organizations that have electronic communication links (other than fax and telephone) with other health services organizations (eg. hospitals, specialized clinic, emergency room, etc). | Administrative data | Canadian Institute for Health Information [1] |
| **10** | **Primary care registries for chronic conditions:**  % of primary care organizations that can produce a list of all patients with diabetes, chronic obstructive pulmonary disease, and cardiovascular disease | Administrative data | Quality and Outcome Framework [4-7] |
| **11** | **Perceived continuity of care[[3]](#footnote-4) :**   - Family physicians’ knowledge of your entire medical history - Other primary care professionals’ knowledge about your medical history and your health - Family physicians’ about the care you receive from other physicians and professionals (for example: visits that you make, treatments recommended)? | Patient reported questionnaire | Primary Care Assessment Survey [2] |
| **12** | **Team work and interdisciplinary care :**   - Perceived effectiveness of primary care professionals’ team effectiveness, leadership and communication - Diversity of professionals within the team - Shared goals, team’s decision and conflict resolution | Clinician or manager reported questionnaire | Team Climate Inventory [8]  Canadian Institute for Health Information [1] |
| **13** | **Links with community organizations:**  Partnerships with community organizations are actively sought to develop formal supportive programs and policies across the entire system | Clinician or manager reported questionnaire | Assessment of Chronic Illness Care [9] |

**TECHNICAL QUALITY OF PREVENTIVE AND CLINICAL CARE**

| **N^o^** | **Quality indicator** | **Source** | **Reference** |
| --- | --- | --- | --- |
| **14** | **Physical activity counselling:**  % of inactive clients/patients who received specific help or information on regular physical activity from their primary care provider, over the past 12 months | Patient reported questionnaire | Canadian Institute for Health Information [1] |
| **15** | **Healthy eating counselling :**  % of clients/patients with unhealthy eating habits who received specific help or information on healthy dietary practices from their primary care provider, over the past 12 months | Patient reported questionnaire | Canadian Institute for Health Information [1] |
| **16** | **Tobacco counselling :**  % of smokers who have received advices or were refered for smoking cessation services | Medical file | Canadian Cardiovascular Outcomes Research Team [10] |
| **17** | **Influenza vaccination:**  % of patients at high risk who are offered influenza vaccination in the last year | Administrative data | European Practice Assessment [11] |
| **18** | **Hypertension screening:**  % of adults whose blood pressure was checked in the last 24 months | Medical file | Canadian Institute for Health Information [1] |
| **19** | **Perceived technical quality of care:**   - Thoroughness of your doctor’s questions about your symptoms and how you are feeling - Thoroughness of doctor’s physical examination to check a health problem you have? - How often do you question whether your doctor’s diagnosis of your health problem is right? | Patient reported questionnaire | Primary Care Assessment Survey [2] |
| **20** | **Clinical management of type 2 diabetes ^4^:**   - % of patients with Type 2 diabetes who have had in the past 15 months: - Their smoking status checked - A measure of their blood pressure - HA1C testing - Microalbuminuria screening - Body mass index measure - A referral for a visual assessment (in the past 24 months) - Foot examination (documented at least once) - % of Type 2 diabetes patients with obesity who have been prescribed metformin or have documented counter-indication or intolerance to metformin | Medical file | Canadian Diabetes Association [12]  Canadian Task Force on Preventive Health Care [13]  Canadian Cardiovascular Outcomes Research Team [10]  Quality Outcome Framework [5]  Canadian Institute of Health Information [1]  National Diabetes Quality Improvement Alliance [14] |
| **21** | **Clinical management of coronary heart disease ^4^:**   - % of adult patients who have: - Their smoking status documented - Their body mass index measured - Their blood pressure recorded on the chart in the past three years - Lipid testing at least every five years recorded on the chart for men 40 to 80 years of age, or women 50 to 80 y.o. - % de patients with ischeamic heart disease who were prescribed, or have documented counter-indication or side-effects to: - A beta-blocker - An acetylsalicylic acid - An angiotensin-converting enzyme inhibitors | Medical file | Canadian Cardiovascular Outcomes Research Team [10]  Canadian Institute of Health Information [1]  Canadian Hypertension Education Program [15] |
| **22** | **Clinical management of chronic obstructive pulmonary disease** **^4^:**  % of patients with chronic obstructive pulmonary disease who have:   - An annual evaluation of their smoking status - An annual evaluation of their respiratory symptoms - Received vaccination against pneumonia - Been prescribed a rapid-acting bronchodilator (stage 1 or more) - Been prescribed a long-action bronchodilator if symptoms are not relieved by as-needed brocondilator (stage 2 or more) - Received teaching on how to use their inhaler device - Been prescribed long-term oxygen if their PaO2 is less than 55mmHg | Medical file | Canadian Cardiovascular Outcomes Research Team [10]  Health Effectiveness Data and Information Set (HEDIS) [16]  Assessing Care of Vulnerable Elders [17]  American Heart Association [18]  Canadian Thoracic Society [19] |
| **23** | **Clinical management of heart failure** **[[4]](#footnote-5):**   - % of patients with heart failure who have: - Their smoking status recorded in the chart - Their weight and cardiac symptoms recorded at each visit - Their blood pressure checked at each visit - Their left ventricular function assessed - % of patients with systolic heart failure who were prescribed, or have documented counter-indication or side-effects to: - A beta-blocker - An angiotensin-converting enzyme inhibitors - % of patients with systolic heart failure who were prescribed warfarin, or have documented counter-indication or side-effects to warfarin. | Medical file | Canadian Cardiovascular Outcomes Research Team [10]  Canadian Cardiovascular Society [20]  American Heart Association [21] |

**INTERPERSONAL RELATIONSHIP**

| **N^o^** | **Quality indicator** | **Source** | **Reference** |
| --- | --- | --- | --- |
| **24** | **Self-care support** **^5^:**  In the past 6 months, chronic disease patients report that they:   - Received a written list of things they should do to improve their health - Were shown how what they did to take care of themselves influenced their condition - Were encouraged to go to a specific group or class to help them cope with their chronic condition - Were helped to plan ahead so that they could take care of their condition even in hard times | Patient reported questionnaire | Patient Assessment of Chronic Illness Care [22] |
| **25** | **Patient participation in clinical decision-making ^5^:**  In the past 6 months, chronic disease patients report that they were:   - Asked to talk about their goals in caring for their condition - Given choices about treatment to think about - Asked about their ideas when making a treatment plan | Patient reported questionnaire | Patient Assessment of Chronic Illness Care [22] |
| **26** | **Respect and empathy [[5]](#footnote-6):**  Chronic disease patients’ satisfaction with the level of attention, empathy, and respect of private life that is demonstrated by their primary care provider | Patient reported questionnaire | Primary Care Assessment Survey [2]  European Task Force on Patient Evaluations of General Practice Care (EUROPEP) [23]  Canadian Institute of Health Information [1] |
| **27** | **Time available during the consultation ^5^:**  Chronic disease patients’ satisfaction with :   - Time available during the consultation - Patience of primary care providers regarding to address their questions and preoccupations | Patient reported questionnaire | Primary Care Assessment Survey [2] |
| **28** | **Trust toward primary care provider ^5^:**  Chronic disease patients report that they have trust in:   - Their primary care provider and hi/her judgement - The fact their primary care provider always tells them the truth - The fact their primary care provider truly cares about their health | Patient reported questionnaire | Primary Care Assessment Survey [2] |
| **29** | **Stress and responsibilities at work and at home ^5^:**  Primary care providers have discussed chronic disease patients’ stress, preoccupations, and responsibilities at work and at home | Patient reported questionnaire | Primary Care Assessment Survey [2]  General Practice Assessment Survey [24]  Agency for Healthcare Research and Quality [25] |

**OUTCOMES**

| **N^o^** | **Quality indicator** | **Source** | **Reference** |
| --- | --- | --- | --- |
| **30** | **Fruit and vegetable consumption rate:**  % of population, 12 years and over, who currently consume five or more servings of fruits and vegetables daily | Population Survey | Canadian Institute of Health Information [1] |
| **31** | **Smoking rate:**  % of population, 12 years and over, who are current smokers | Population Survey | Canadian Institute of Health Information [1] |
| **32** | **Physical activity rate:**  % of population, 12 years and over, who currently engage in regular physical activity | Population survey | Canadian Institute of Health Information [1] |
| **33** | **Blood pressure control:**  % of patients identified as hypertensive for longer than 12 months whose most recent blood pressure was at target:   - Below 140/90 for nondiabetics - Below 130/80 for diabetics or patients with renal disease - Below 125/75 for patients with proteinuria | Medical file | Canadian Cardiovascular Outcomes Research Team [10] |
| **34** | **Perceived self-efficacy:**  Chronic disease patients trust in their ability to:   - Limit the impact of fatigue, pain, and emotional distress caused by their disease on their daily life - Do the different activities needed to manage their health condition and reduce their need to see a doctor - Do things other than taking medication to reduce the impact of illness on their daily life | Patient reported questionnaire | Standford Self-Efficacy 6-item Scale [26] |
| **35** | **Hospitalisation for ambulatory care sensitive conditions:**  Age standardized acute care hospitalization rate for conditions where appropriate ambulatory care prevents or reduces the need for admission to hospital in population 75 years and under:   - diabetes - angina - chronic obstructive pulmonary disease - heart failure   (Excluding cases where inter-hospital transfer or death occurs during admission) | Administrative data | Canadian Institute of Health Information [1] |
| **36** | **Emergency room visits for ambulatory care sensitive conditions:**  % of primary care patients, ages 20 to 75 years, with congestive heart failure, diabetes, COPD, or angina who visited the emergency department for a decompensation/exacerbation of their condition in the past 12 months | Administrative data | Canadian Institute of Health Information [1] |
| **37** | **Quality of life:**  Chronic disease patients’ perception of the impact of their chronic condition on their quality of life | Patient reported questionnaire | Minnesota Living with Heart Failure Questionnaire [27]  Chronic respiratory Disease Questionnaire [28]  Audit of Diabetes-Dependent Quality of Life [29]  The Medical Outcomes Study Short Form 36 (SF-36) [30] |

## Original indicator sets

1. The PCAS survey is framed around the perceived difficulty to obtain an appointment at the doctor’s office. This item has been adapted to refer to primary health care providers, including family physicians and primary care nurses. [↑](#footnote-ref-2)
2. Excluding access to “Info-Santé”, a provincial government service providing generic health advices over the phone. [↑](#footnote-ref-3)
3. “Regular doctor” was changed to “family physician” [↑](#footnote-ref-4)
4. Participants were informed that they would be allowed to narrow down the specific indicators measured within each disease-specific clinical indicator item, should they choose it as target for improvement. [↑](#footnote-ref-5)
5. “Doctor” in the original indicator set has been changed for “primary care provider” [↑](#footnote-ref-6)
